# Improvement in hypertrophic cardiomyopathy after using a high-fat, high-protein and low-carbohydrate diet in a non-adherent child with glycogen storage disease type IIIa

**DOI:** 10.1016/j.ymgmr.2022.100904

**Published:** 2022-08-01

**Authors:** Burcu Kumru Akin, Burcu Ozturk Hismi, Anne Daly

**Affiliations:** a Division of Nutrition and Diet, Gaziantep Cengiz Gökçek Maternity and Children's Hospital, Gaziantep, Turkey; b Division of Pediatric Metabolic Disorders and Nutrition, Marmara University School of Medicine, Istanbul, Turkey; c Birmingham Women's and Children's Hospital, NHS Foundation Trust, Birmingham B4 6NH, UK

**Keywords:** Glycogen storage disease type III, Hypertrophic cardiomyopathy, Diet therapy

## Abstract

**Background:**

Glycogen storage diseases type IIIa and b (GSDIII) are rare inherited metabolic disorders that are caused by deficiencies of the glycogen debranching enzyme, resulting in the accumulation of abnormal glycogen (‘limit dextrin’) in the muscles. The cardiac storage of limit dextrin causes a form of cardiomyopathy similar to primary hypertrophic cardiomyopathy. Treatment with a high fat diet is controversial but we report a positive outcome in a child with cardiomyopathy.

**Case presentation:**

A 9-year-old boy with GSDIIIa developed left ventricular hypertrophy at 4.3 years of age. A high-fat (50%), high protein (20%), low-carbohydrates (30%) diet was introduced. After 18 months, echocardiogram, biochemical and clinical parameters improved (Creatine Kinase (CK), 1628➔1125 U/L; left ventricular outflow tract (LVOT), 35➔20 mmHg; interventricular septum (IVS), 21➔10 mm). The diet was abandoned for 2 years resulting in reversal of symptoms, but recommencement showed improvement after 6 months.

**Conclusion:**

A high fat, high protein and low carbohydrate diet was successful in reversing cardiomyopathy. This form of treatment should be considered in children with GSD IIIa with cardiomyopathy.

## 1 Introduction

GSDIIIa and b, are disorders of glycogenolysis, caused by mutations in the glycogen debranching enzyme (amylo α1,6-glucosidase, 4α glucanotransferase) encoded by the AGL gene. This debrancher enzyme cleaves glucose at 1–6 α linkages, and its absence results in incomplete breakdown of glycogen, hypoglycaemia and the formation of limit dextrin. Limit dextrin is an abnormal form of glycogen accumulating between the myofilament bundles in muscles [1], and leads to hypertrophic cardiomyopathy, which may progress to asymptomatic left ventricular hypertrophy [2,3]. In a review by Sentner et al. [4], from 175 patients with GSDIIIa in which 52% had reached adulthood, cardiac involvement was reported in 58% (n = 87/151), and cardiomyopathy in 15% (n = 22/151) this was not accompanied by biochemical parameters of raised cholesterol, or triglycerides.

GSD IIIa has an approximate prevalence ±85% with in the group of GSD III's affecting the breakdown of glycogen in the liver, heart and skeletal muscle, while type b has an estimated prevalence of ±15% with in the group of GSD III's only affecting liver glycogenolysis [5,6]. Reversal of type IIIa, cardiomyopathy has previously been described using a high protein diet with or without a high fat diet [2,7,8], but there is no consensus on dietary treatment. This view has been challenged with reversal of cardiomyopathy using a high fat, high protein and low carbohydrate diet [8,9], which, also remains controversial with a lack of data to support its use. Not all children with type IIIa GSD develop cardiac difficulties, but for those who do effective treatment is essential.

Traditionally dietary management aims to maintain normoglycaemia by using sufficient carbohydrate and additional protein providing gluconeogenic amino acids as a substrate for glucoenogensis together with uncooked cornstarch [10]. Hepatomegaly, failure to thrive, ketotic hypoglycemia and elevated transaminases are observed in patients with GSDIII [4]. To prevent hypoglycaemia some centres use overnight tube feding although practices vary between centres and countries [11]. Achieving normoglycemia is important, although this is not sufficient to prevent long-term complications such as myopathy and cardiomyopathy [10]. Myopathy progresses slowly, often manifesting in exercise intolerance motor delay and fatigue [12]. There is no consensus on the optimal dietary treatment to prevent this long-term co morbidity.

The GSDIII consensus guidelines published by the American College of Medical Genetics recommend high protein (25% of total calories), low complex carbohydrates (<50% of total calories) and avoidance of simple sugars [13]. In recent case reports, it has been observed that a range of dietary therapies have been studied: including ketogenic diets, modified Atkins diet, high protein, high fat, medium chain triglyceride (MCT) and synthetic ketone bodies [2,[7], [8], [9],[14], [15], [16]]. Each has met with a range of success in reversing cardiomyopathy and maintaining normoglycaemia.

This case report presents the case of a Turkish boy with GSDIIIa whose hypertrophic cardiomyopathy resolved completely upon starting a high-protein, high-fat and low-carbohydrate diet. Hypertrophic cardiomyopathy re-occurred after he was lost to follow-up and abandoned the diet for two years, but resolved after the reintroduction of the same diet.

### 1.1 Case presentation

**Fig. 1:**
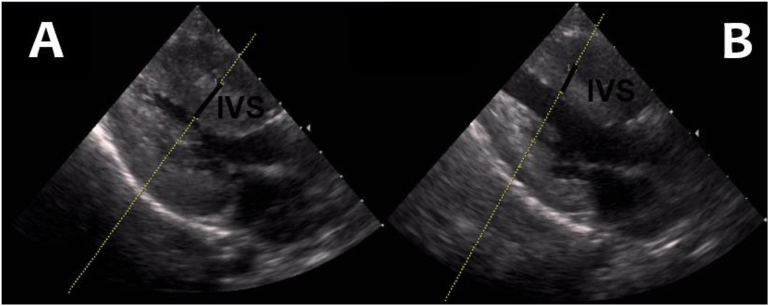
Echocardiographic findings in a patient with glycogen storage disease type III upon detection of hypertrophic cardiomyopathy before therapy (A) and 18 months after the introduction of a high-fat and low-carbohydrate diet (B); *IVS,* interventricular septum.

GSD IIIa was diagnosed in a male patient, the third child of consanguineous parents, at the age of 4 months. *AGL* sequencing, showed a homozygous pathogenic c.1783C > T variant (HGMD No: CM098740) leading to a stop codon (p.Arg595*). He presented with hypoglycaemia, ketotic hypoglycemia, hepatomegaly, elevated serum creatine kinase (CK) levels, fatigue and exercise intolerance at the age of 3 years and 9 months. A traditional high protein diet and uncooked cornstarch had been introduced, but the early dietetic and clinical history was not provided as he was treated at another hospital. On presentation he was failing to thrive and stunted weight-for-height *z*-score: 0.52; height-for-age *z*-score: −2.28, had poor oral hygiene, hepatomegaly, and grade I/VI systolic hearth murmur. Estimated energy intake from parental history was 1200 Kcal/day, 15% lower than the estimate average requirement for age. The family had abandoned dietary treatment. His fasting tolerance was estimated to be 5 h although this was not formally tested. An electrocardiogram showed signs of left ventricular hypertrophy, and echocardiography showed hypertrophic cardiomyopathy, along with an increased end-diastolic interventricular septum, left ventricular posterior wall thickness and left ventricular outflow tract (LVOT) obstruction (Fig. 1).

As a result of the cardiomyopathy and suggested evidence from Brambilla et al. [9] a high fat high protein and low carbohydrate diet was introduced: 50% fat, 20% protein and 30% carbohydrate providing 1400 kcal/day. Home blood glucose monitoring was arranged, and cornstarch Glycosade® (Vitaflo International Ltd. Liverpool, UK) providing 2 g/kg/day was recommended to be taken 4 times per day to prevent hypoglycaemia. Dietary fat was provided from unsaturated fatty acids as olive oil. Additional protein to meet the dietary prescription was provided from Protifar® (Nutricia, Fulda, Germany) which has a composition of 20% casein 80% whey providing 1 g/kg/body weight.

Regular clinical follow showed improved biochemical and echocardiograms. Fatigue and weakness abated after 18 months of diet therapy; exercise capacity was reported to be increased, (according to family observation) and cardiac imaging improved although growth remained suboptimal weight-for-height *z*-score: −0.53; height-for-age *z*-score: −2.23 (Table 1 and Fig. 1).

**Table 1:** Time of diagnosis of clinical, biochemical and echocardiographic findings at baseline and after therapy.

Parameters (Time)	Diagnosis	Baseline (0 m)	18 months later (18 m)	Follow-up 2 years with no diet therapy later (42 m)	6 months later (48 m)
*Clinical findings*					
Hypoglycemia	+	+	−	−	−
Hepatomegaly	+	+	+	+	+
Growth failure		+	−	+	−
Cardiomyopathy	−	+	−	+	−
*Diet composition*					
Carbohydrate (%)		48	30	60	30
Protein (%)		15	20	10	20
Lipid (%)		37	50	30	50
*Biochemical data*					
CK (U/L)	667	1628	1125	2493	2289
AST (U/L)	134	306	1099	425	426
ALT (U/L)	136	319	724	402	403
TG (mg/dl)	377	186	291	210	316
*Echocardiographic monitoring*					
IVS (mm)	Normal	21	10	16	12
PW (mm)	Normal	18	11	7.9	9.5
LVOT gradient (mmHg)	Normal	35	20	60	25

ALT, alanine aminotransferase; AST, aspartate aminotransferase;, creatine kinase; IVS, interventricular septum; LVOT, left ventricular outflow tract; PW, posterior wall thickness; TG, triglyceride.

Unfortunately after successful treatment of the hypertrophic cardiomyopathy, the family were lost to follow up, and he was re-admitted due to weakness and fatigue at the age of 8.5 years, he was stunted; weight-for-height *z*-score: −1.54; height-for-age *z*-score: −2.07. The patient had abandoned dietary treatment following a high-carbohydrate, low-fat and low-protein diet and had subsequently developed hypertrophic cardiomyopathy, although in a milder form than in the previous diagnosis. The diet history showed non adherence to the prescribed diet and at 8 years of age there was a reluctance to accept any dietary changes. Echocardiographic, biochemical and clinical findings improved 6 months after the introduction of a high-fat and high-protein diet at home. Unfortunately, after improved of hypertrophic cardiomyopathy, the patient was lost to follow up.

Biochemical and echocardiographic findings at baseline and after the introduction of a high-fat and low-carbohydrate diet are presented in Table 1 and Fig. 1. These show that effect of diet therapy on biochemical and echocardiographic findings.

## 2 Discussion

This case study demonstrates that treatment with a high fat high protein and low carbohydrate diet successfully reversed hypertrophic cardiomyopathy in a non-compliant family. Recent studies suggest that patients with GSDIIIa have a higher fat and protein requirement than normal children as a result of the increased rate of *gluco* and ketogenesis^(^ [9,15,16)]. It is suggested that the inability to satisfy this higher energy demand and the storage of excess carbohydrate in the form of dextrin may play a role in the pathogenesis of hypertrophic cardiomyopathy [9]. However there remains an unanswered question: what is the optimal therapeutic diet for resolving cardiomyopathy in children with GSDIIIa?

Although there is a growing amount of evidence to suggest a high fat ± high protein and low carbohydrate diet is efficacious in the treatment of GSD type IIIa, the studies to date are mainly case reviews with a small number of subjects, variable ages and a heterogeneous group of adults and children, making any robust conclusion difficult. Despite these limitations there is clearly an improvement in cardiac function on dietary intervention and this cannot be dismissed.

Table 2 describes 7 case studies from 9 GSDIII subjects who developed cardiomyopathy. Of these patients, 22% (n = 2) were treated with a high protein diet, 22% (n = 2) with a high-fat diet, and 56% (n = 5) with a high-fat and high-protein diet. Synthetic ketone bodies were used in only one patient age 2 months old. Cardiomyopathy improved in all patients and CK levels decreased in 56% (n = 5) of patients. Among the patients with decreased CK levels, 3 patients were on a high-fat and high-protein diet, 1 patient was on a high protein diet, and 1 patient was on a high-fat diet. These results are mixed, all dietary interventions improved cardiac function and CK was decreased in half the reported cases regardless of dietary intervention. All have one common denominator being low in carbohydrate.

**Table 2:** Summary of publications on dietary intervention and outcomes in GSDIII.

Author/year	Number of patients/age	Dietary treatment	Outcome
Dagli [7]	1 (23 years)	Protein: 30%	Cardiomyopathy improved
		Lipid: saturated and carbohydrate: 70%	CK levels decreased
Valayannopoulous [8]	1 (2 months)	Protein: 15%	Cardiomyopathy improved
		Lipid: 65% (with synthetic ketone bodies)	Insulin and CK levels decreased
		Carbohydrate: 20%	
Sentner [2]	1 (32 years)	Protein: 37% to 43%	Cardiomyopathy improved
		Lipid: 2%	Body mass index decreased
		Carbohydrate: 61%	
Mayorandan [15]	2 (9, 11 years)	Protein: 7 g/kg/per day	Cardiomyopathy improved
		Lipid: 8 g/kg/per day	CK levels decreased
		Carbohydrate: 0.4 g/kg/per day	
Brambilla [9]	2 (5, 7 years)	Protein: 25%	Cardiomyopathy improved
		Lipid: 60%	CK levels decreased
		Carbohydrate: 15%	
Francini-Pesenti [16]	1 (34 years)	Protein and lipid: allowed ad libitum with olive oils and medium chain triglyceride	Cardiomyopathy improved
		Carbohydrate: limited to 20 g/per day	CK levels decreased
Marusic [14]	1 (15 years)	Protein: 11%	
		Lipid: 87%	Cardiomyopathy improved
		Carbohydrate: 2%	

One hypothesis that may account for the cardiac improvement are elevated ketone bodies and fatty acids, which are the preferred energy substrates for the heart and skeletal muscle, possibly improving muscles performance [17]. Rossi et al. [18] in a systematic review describes 28 GSDIII patients with associated cardiomyopathy / myopathy who were treated with a high fat diet. In pediatric GSDIIIa patients a decrease in CK concentrations (n = 7, *p* < 0.01) and a reduction in cardiac hypertrophy (n = 19, p < 0.001) were observed after starting a high fat diet. However, a link between high cholesterol and triglycerides in GSD III has been associated with an increased risk of osteoporosis combined with reduced bone mineral density [19,20]. Although bone mineral density was also linked to decreased serum levels of growth hormone, insulin and bone makers osteocalcin and C terminal cross linked telopeptide (CTX). A ketogenic diet may improve myopathy in patients with GSDIII, as ketones are used as an alternative energy source, although such a diet may also cause deterioration in liver inflammation and may be associated with growth retardation [21]. The experience with increased dietary fat and the use of Atkins and ketogenic diets relies on case reports and expert opinions [21].

The weight of evidence to support the efficacy of a high-fat diet in pediatric GSDIIIa patients with cardiac hypertrophy appears to be increasing, but careful long-term follow-up is required as potential complications such as growth restriction, bone health, liver inflammation, hepatosteatosis, hepatic fatty liver disease, and hepatocellular carcinoma need to be considered [18,22]. Concurrently, an increased protein intake may equally be efficacious by improving muscle function and increasing muscle protein synthesis. One theory to account for this improvement is via the mechanistic target of rapamycin (mTOR). In muscle tissue there is a balance between muscle synthesis and breakdown, mTOR plays a critical role in regulating protein synthesis. It is unknown if this delicate synergy is disturbed in GSDIII and research is needed to understand the role of mTOR and protein synthesis in this group of patients [23].

The common denominator in all these treatments is a low refined carbohydrate intake. A low carbohydrate diet is associated with reduced hyperinsulinism and suppressed lipolysis, keto and gluconeogenesis and the activation of glycogen synthesis [15]. Simple sugars lead to rapid swings in blood glucose levels [13], leading to excess glycogen stored in the liver increasing hepatomegaly. Cornstarch should be given in measured amounts preventing hypoglycaemia avoiding excessive storage of glycogen and the development of insulin resistance and obesity [21,24].

Any therapeutic diet should contain sufficient energy and micronutrients to meet requirements for growth. Insufficient energy reduces muscle function leading to severe exercise intolerance and/or signs of hypoglycemia during exercise [25]. Beta-blocker therapy may be prescribed for hypertrophic cardiomyopathy but needs careful monitoring as they may mask the symptoms of hypoglycemia [13].

In conclusion, the current case report presents a child with GSDIIIa treated with a high-protein, high-fat, and low-carbohydrate diet. The improvement in cardiomyopathy both after the initial diagnosis and after the re-introduction of diet therapy is evidence of a successful dietary intervention. The recurrence of cardiomyopathy after the diet therapy was abandoned suggests that diet therapy should be long term, although there is no clear data regarding specific durations. There are still many questions that need to be answered, such as why some patients develop myopathy/cardiomyopathy while others do not, and it is unknown how these clinical manifestations are affected by the genotype, age or type of diet therapy.

There is clearly a need for further studies to bring clarity on the mechanisms of using a high protein ± high fat with a low carbohydrate intake in this vulnerable group of children and adults. Optimal intakes of protein, fat and carbohydrate are as yet unknown and collaboration is needed to bring clarity on optimal dietary management preventing long term co-morbidity and cardiac symptoms.

## 3 Conclusion

Unfortunately, we could not see the long-term effects of diet therapy, which was started for the second time, as the patient failed to engage with clinical treatment. However, dietary intervention was successful on two occasions resolving the cardiac symptoms.

## Funding

No funding has been received for this work.

## Author contributions

B.K.A. analyzed the data and wrote the first draft; B.K.A., B.O.H. cared for the patient, collected clinical data and analyzed the results; B.K.A. organized the dietary management; B.O.H. overviewed the clinical and nutritional management; B.K., B.O.H. and A.D. prepared the final version of the manuscript.

## Declaration of Competing Interest

No conflict of interest was declared.

## Data Availability

The data that has been used is confidential.
